# Molecular characterization of heterogeneity in adult T-cell leukaemia/lymphoma

**DOI:** 10.1186/1742-4690-12-S1-P69

**Published:** 2015-08-28

**Authors:** Huseini Kagdi, Aileen Rowan, Maria Antoinietta Demontis, Charles Bangham, Graham Taylor

**Affiliations:** 1 Section of Retrovirology and GU medicine, Department of Medicine, Imperial College London, London, England, W2 1PG UK; 2 Section of Virology, Department of Medicine, Imperial College London, London, England, W2 1PG UK

## 

Adult T cell leukaemia/lymphoma (ATL) is a highly heterogeneous neoplasm caused by human T cell lymphotropic virus type 1 (HTLV-1) infection. Shimoyama et al first proposed a prognostic classification based on clinical features. However subsequent studies have highlighted even greater heterogeneity and progression from indolent to aggressive subtypes. The molecular basis for this heterogeneity and progression remains unknown. Our aims were to identify molecular markers that characterize this heterogeneity and progression. We studied expression of T-cell markers and clonality (Ligation Mediated PCR) on peripheral blood mononuclear cells in samples collected from HTLV-1 infected patients (16 asymptomatic carrier [AC], 18 with HTLV-1-associated myelopathy [HAM], 18 with indolent ATL [five smouldering & 13 chronic] and seven with acute ATL) attending the National Centre for Human Retrovirology, London. 11/18 indolent ATL required no systemic treatment and one progressed to acute ATL. The remainder required systemic therapy and all except one achieved complete remission with zidovudine/interferon. 6/7 with acute ATL had primary refractory disease. Clonality testing showed no dominant clones in the AC, patient with HAM and 6 of the 11 patients with indolent ATL who didn't require systemic therapy. We confirmed CD4+CCR4+ as the main reservoir of HTLV-1 infection. These CD4+CCR4+ T cells had CD7+CD25+CD26+CD127+CCR7-immunophenotype in AC, HAM and indolent ATL with no dominant clones. CD7 down-regulation within CD4+CCR4+ T cells identified ATL patients with dominant clone(s). CD127 down-regulation within CD4+CCR4+ T cells was present in all indolent ATL patients which required systemic therapy. Nine patients with ATL (all acute and two indolent) had CCR7 up- regulation of which all but two had primary refractory disease. Longitudinal follow up in six patients showing changing immunophenotype which correlated with clinical features and prognosis. In conclusion CD7, CD127 and CCR7 expression within CD4+CCR4+ infected cells further characterises the heterogeneity within ATL with therapeutic implications.

